# Graphene oxide-engineered dental implants: orchestrating antibacterial defense and osteo-immunomodulation for enhanced osseointegration

**DOI:** 10.3389/fbioe.2026.1943704

**Published:** 2026-09-23

**Authors:** Qingfan Li, Yun Wang, Jiahui Hu, Zuolin Wang

**Affiliations:** 1 School of Stomatology, Tongji University, Shanghai, China; 2 Tongji Research Institute of Stomatology, Shanghai Engineering Research Center of Tooth Restoration and Regeneration, Shanghai, China; 3 Anhui Provincial Key Laboratory of Oral Diseases Research, College of Stomatology, Anhui Medical University, Hefei, China

**Keywords:** antibacterial, dental implant, GO, macrophages, osteoimmunomodulation

## Abstract

Dental implant technology has brought significant benefits to millions of patients with missing teeth, substantially improving their quality of life. However, titanium-based dental implants still face two stubborn problems—peri-implant infection and inadequate osseointegration, which seriously undermine their therapeutic outcomes and overall clinical success. To address these two key bottlenecks, this study developed a graphene oxide (Graphene oxide)-engineered dental implant endowed with synergistic antibacterial and osteoimmunomodulatory properties. This implant exhibited broad-spectrum antibacterial effects against three common pathogenic strains in dentistry by disrupting bacterial cell membranes and increasing intracellular reactive oxygen species (ROS) levels. Moreover, the implant regulated the macrophage polarization toward a pro-regenerative phenotype, thereby optimizing the local immune microenvironment and promoting the extension and differentiation of osteoblasts. *In vivo* studies confirmed that GO-engineered implants enhanced antibacterial defenses, promoted inflammation resolution, and accelerated osteogenic regeneration. These results suggest the preclinical potential of GO-based surface modification for dental implants. By integrating antibacterial, immunomodulatory, and osteogenic functions, this implant demonstrates promising preliminary performance in promoting early osseointegration and short-term local biological stability, offering a candidate strategy for further implant design.

## 1 Introduction

According to statistics, approximately two billion people worldwide are troubled by various oral health problems, resulting in their tooth loss. In modern dentistry, titanium-based implants have been widely adopted in clinical practice, providing a solution for both aesthetic restoration and functional recovery for patients with partial or complete tooth loss (Wang et al., 2021a; Remísio et al., 2023; Wang et al., 2021b; Souza et al., 2019).

Despite their widespread application of clinical titanium-based implants, two critical challenges remain to be effectively addressed: peri-implant infections and the optimization of osseointegration efficiency. Bacterial colonization-induced implant infections often impair osseointegration, which represents a primary cause of failure in titanium-based dental implants (Silva et al., 2023). Although postoperative antibiotic therapy is widely used to manage bacterial infections, prolonged antibiotic administration may lead to the emergence of antimicrobial resistance (Wang et al., 2018). Furthermore, antibiotic treatments fail to prevent the persistent infection risk that may occur during the extended postoperative period after implantation or prosthetic restoration. Consequently, an ideal implant material should possess dual-phase antibacterial efficacy, characterized by both immediate and sustained broad-spectrum antibacterial activity. Immediate antibacterial activity refers to a rapid contact-killing effect occurring within minutes to hours upon bacterial exposure, which is critical for preventing initial bacterial colonization during and immediately after surgery. Sustained antibacterial activity refers to a durable antibacterial effect persisting over days to weeks, which is essential for preventing late-stage infections during the extended postoperative healing period. In addition, osteoimmunomodulation around the implant is crucial for achieving osseointegration, which underscores the necessity of incorporating osteo-immunomodulation strategies into implant design (Ouyang et al., 2021). Following device placement, macrophages rapidly migrate to the implant-bone interface, where their behavior substantially determines the success of the implant (Zhao et al., 2022). Upon homing to the interface sites, these immune cells are activated and polarize into distinct functional phenotypes—the classically activated pro-inflammatory phenotype (M1) and the alternatively activated pro-healing phenotype (M2) (Albrektsson et al., 2023). Therefore, implant-based osteoimmunomodulatory strategies have demonstrated the capacity to modulate macrophage polarization from the pro-inflammatory state toward a pro-healing state, thereby enhancing osseointegration (Chen et al., 2024; Li et al., 2020a; Li et al., 2020b).

Graphene oxide (GO) has attracted significant attention due to its potential in biomedical applications, especially in bone regeneration (Niknam et al., 2022; Derakhshi et al., 2022). For instance, composite materials incorporating GO with calcium phosphate nanoparticles have been found to significantly enhance osteogenic differentiation of human mesenchymal stem cells (hMSCs) while accelerating mineralization processes (Tatavarty et al., 2014). Moreover, GO exhibits prominent antibacterial properties (Pandit et al., 2021). A comparison and evaluation of antibacterial effects of graphene and its derivatives was conducted using *E. coli* (*Escherichia coli*) under uniform concentration and culture conditions, and revealed that GO exhibited the strongest antibacterial activity (Liu et al., 2011). Furthermore, GO could inhibit the growth of *E. coli* by exceeding 90% within 2 hours (Hu et al., 2010). This antibacterial performance was attributed to physical disruption of bacterial membranes by the sharp nanostructures of GO. Additionally, other researchers have reported that GO-zinc oxide nanocomposites exert inhibitory effects on *Streptococcus mutans* aggregated in biofilms on implant surfaces (Kulshrestha et al., 2014). In addition, GO has been found to elicit an immunomodulatory response that can affect the macrophage phenotype (Ma et al., 2015). GO maintained the M1/M2 balance by transforming to the M2 pro-healing phenotype, thereby ensuring an appropriate immune response (Feito et al., 2019). These outstanding characteristics indicate that GO holds great potential for implant surface modification to regulate macrophage responses and resist bacterial infection at the implant-tissue interface.

Based on the aforementioned rationale, we developed a GO-engineered titanium implant consisting of a titanium SLA substrate and a GO coating. The GO coating is the key functional component that confers both broad-spectrum antibacterial activity and osteoimmunomodulatory properties. Specifically, this implant exhibits three significant characteristics: (1) effective broad-spectrum antibacterial capability, (2) enhanced *in vitro* osteogenic gene expression and matrix mineralization through optimized osteoimmune regulation, and (3) improved *in vivo* osseointegration. By simultaneously addressing antibacterial and immunomodulatory requirements via the GO coating, this system offers substantial clinical value for dental implant design and application.

## 2 Materials and methods

### 2.1 Fabrication of GO-Engineered titanium substrates

Grade IV pure titanium plates (10 × 10 × 1 mm) underwent sandblasting using Al_2_O_3_ particles at 5 bar pressure, with a 10 cm nozzle-to-substrate spacing. Subsequent acid etching was carried out in an HCl–H_2_SO_4_ mixture at 60 °C for 8 h to generate large-particle sandblasted and acid-etched surfaces (SLA, the currently predominant method for modifying dental implants in clinical practice). To impart surface amine functionality, the SLA-treated substrates were incubated in a 3% APTES solution for 1 h. GO deposition on the SLA-treated substrates was achieved through ultrasonic atomization spraying (Siansonic equipment). Operational parameters included: 250 μL/min GO dispersion flow rate, 10 cm nozzle-to-surface distance, and 90 s spraying duration. All modifications employed 0.05% (w/v) GO solutions according to the guidelines (Li and Wang, 2020). Post-treatment cleansing involved sequential rinsing with anhydrous ethanol and ultrapure water. Control groups comprised SLA-only specimens (designated SLA group), while GO-modified counterparts were termed SLA/GO group (Supplementary Figure 1A; Supplementary Figure 1B). For *in vivo* biological evaluation, micro-implants (2.2 mm in diameter and 3.5 mm in length, 0.35 mm thread depth, 0.7 mm pitch) featuring identical SLA or SLA/GO surface treatments were fabricated (Supplementary Figure 1C; Supplementary Figure 1D).

### 2.2 Surface characterization of GO-Engineered titanium substrates

The surface properties of the modified substrates were comprehensively characterized using multiple analytical techniques. Before this, the transmission electron microscope (TEM; JEM-F200, JEOL, Japan) was applied to scan the nanostructure of GO. Then, surface morphology was examined by field emission scanning electron microscopy (FE-SEM; S-4800, Hitachi, Japan) operating at an acceleration voltage of 5.0 kV. Topographic features and surface roughness were quantified by atomic force microscopy (AFM; NanoScope MultiMode IIIa, Veeco, USA) in tapping mode. Chemical bonding characteristics were determined through Raman spectroscopy (LabRAM, Horiba Jobin Yvon, France) employing a 532-nm Ar-ion laser with 20 mW power, scanning the spectral range of 1,000–3,000 cm^-1^. For chemical state and elemental composition analysis, X-ray photoelectron spectroscopy (XPS; PHI 5000C ESCA system, PHI, USA) was utilized. Surface wetting property was evaluated using a contact-angle goniometer (DSA100, Krüss, Germany) to determine hydrophilic/hydrophobic properties.

### 2.3 Bacterial culture

The bacterial strains were cultivated under specific conditions: *Staphylococcus aureus* (*S. aureus*, ATCC 6538) was grown in Luria-Bertani medium (Hopebio) at 37 °C containing 5% CO_2_. For anaerobic strains, *P. gingivalis* (*Porphyromonas gingivalis*, ATCC 33277) and *F. nucleatum* (*Fusobacterium nucleatum*, ATCC 25586) were cultivated in brain heart infusion (BHI, Hopebio) supplemented with essential nutrients, including 1 mg/L vitamin K_1_ (Hopebio), 0.5 g/L L-cysteine hydrochloride (Solarbio), 5 g/L yeast extract (Solarbio), and 5 mg/L hemin (Hopebio), and were cultured in an anaerobic incubator at 37 °C, with a gas mixture of 80% N_2_, 10% H_2_, and 10% CO_2_. Solid media were prepared using BHI agar containing menadione, hemin, and 5% defibrinated sheep blood (Solarbio).

### 2.4 *In Vitro* antibacterial activity assessment

The antibacterial efficacy of the tested materials was evaluated using a plate count assay. Bacterial suspensions (1 × 10^6^ colony-forming units (CFU) mL^-1^) of 1 mL were incubated with each substrate for 2 h. After being rinsed with PBS three times, 1 mL of PBS was added to each substrate, and they were vortexed for 2 min and gently sonicated for 3 min to detach the bacteria from the substrates. Then, the collected bacteria were subjected to a series of dilutions, and plated for cultivation. To ensure the homogeneity of the bacterial suspension before plating, it was vigorously vortexed for an additional 10 s to disperse remaining bacterial clumps before each dilution. Additionally, to prevent local accumulation during the spreading process, all agar plates were pre-dried for 15 min before plating to remove surface moisture. Photograph the plates and count the colonies. Subsequently, the antibacterial rate was calculated by comparing the bacterial colonies of the SLA/GO surface with those of the SLA control surface. A double-stained fluorescence microscope (with live and dead bacteria fluorescing green and red, respectively) was used for observation, and quantitative analysis was conducted using ImageJ software.

The bacterial outer membrane permeabilization induced by the samples was evaluated using an ortho-nitrophenolgalactoside (ONPG) hydrolysis assay. Bacterial suspensions (1 × 10^6^ CFU mL^−1^) of 1 mL were exposed to each substrate and incubated for 12 h. Then, 100 μL aliquots from each group were combined with 50 μL of ONPG detection reagent (Beyotime Biotechnology, China), followed by a 30-min dark incubation. The enzymatic hydrolysis of ONPG was quantified by measuring the absorbance at 420 nm using a microplate reader.

To determine intracellular adenosine triphosphate (ATP) levels, bacterial suspensions (1 × 10^6^ CFU mL^−1^) of 1 mL were incubated with the samples for 12 h. After that, 100 μL of lysis buffer was introduced, and the mixture was centrifuged at 12,000 g (4 °C, 5 min). The supernatant (50 μL) was then mixed with 100 μL of ATP detection solution (Beyotime Biotechnology, China) and allowed to react for 3–5 min. Chemiluminescence signals were recorded employing a fluorescence microplate reader (BioTek, Vermont, USA).

Reactive oxygen species (ROS) of bacteria were identified using 2′,7′-dichlorofluorescein diacetate (DCFH-DA). Bacterial suspensions (1 × 10^6^ CFU mL^−1^) of 1 mL were exposed to each substrate and incubated for 12 h and then the supernatant was treated with 100 μM DCFH-DA for 1 h. The formation of ROS was quantified by fluorescence spectroscopy applying an excitation/emission wavelength pair of 485 and 528 nm.

### 2.5 Cell culture and preparation of macrophage-conditioned medium

Murine macrophage cell RAW 264.7 (ATCC) was cultured and maintained in Dulbecco’s modified Eagle’s medium (DMEM; Gibco, USA) containing 10% fetal bovine serum (FBS; HyClone, USA) and 1% penicillin-streptomycin (Gibco, USA), under standard culture conditions (37 °C, 5% CO_2_). Meanwhile, murine osteoblasts MC3T3-E1 (ATCC) were grown in α-minimum essential medium (α-MEM) supplemented with 10% FBS and 1% penicillin-streptomycin under identical incubation conditions. For osteogenic differentiation, the α-MEM basal medium was further enriched with 10 mM β-glycerophosphate disodium salt and 50 μg/mL ascorbic acid to prepare osteogenic induction medium (OM).

To generate macrophage-conditioned medium (CM), RAW 264.7 cells were plated on designated substrates for 24 h, followed by stimulation with lipopolysaccharide (LPS; 1 μg/mL). The supernatant was harvested daily over a 7-day period and subsequently mixed at a 1:1 ratio with fresh osteoblast culture medium to obtain the final CM.

### 2.6 *In Vitro* osteoimmunomodulatory assessment

To evaluate the cytotoxicity and cell viability of the material, flow cytometry (FCM) was conducted at 1, 3, and 7 days after cell culture on distinct substrate surfaces. Cells were collected and resuspended in 400 μL binding buffer. Then, 10 μL of Annexin V and 5 μL of propidium iodide (PI) staining solution were added and incubated in the dark for 30 min; subsequently, flow cytometry (FACSVerse, BD Biosciences, USA) analysis was performed. Data analysis was conducted using FlowJo 7.6 software, and a two-color dot plot was prepared (Annexin V as the X-axis and PI as the Y-axis).

Macrophages were cultured on distinct substrate surfaces to detect the effect of GO modification on macrophages (Supplementary Figure 2A). For analysis of macrophage polarization markers, FCM was employed to evaluate surface protein expression. Cells were grown on each substrate for 3 and 7 days, followed by staining with primary antibodies (FITC-CD11c, PE-CD86, PE-CD163, and APC-CD206; BioLegend, USA) for 1 h and secondary antibodies for 30 min under dark conditions. After fixation, cells were analyzed using a flow cytometer. For flow cytometric analysis, the macrophages were first gated based on forward scatter (FSC) and side scatter (SSC) characteristics to exclude debris and doublets. Single cells were then gated for CD11 b^+^ to identify the macrophage population, followed by analysis of M1 (CD86^+^ and CD11c^+^) and M2 (CD163^+^ and CD206^+^) markers. Finally, the data were processed with FlowJo 7.6 software.

The mRNA levels of inflammation-associated genes (TNF-α, iNOS, IL-10, and Arg-1) in macrophages were quantified through reverse transcription-polymerase chain reaction (RT-PCR). Murine macrophages were maintained on distinct substrate surfaces for 3 and 7 days. Total RNA isolation was performed applying TRIzol reagent (Roche, Switzerland), followed by cDNA synthesis with the Transcriptor First Strand cDNA Synthesis Kit (Roche, Switzerland). Quantitative PCR amplification was carried out with FastStart Universal SYBR Green Master Mix (Roche, Switzerland) on a LightCycler® 96 system (Roche, Switzerland). The relative expression of target genes was quantified by the 2^−ΔΔCT^ method, using Gapdh for normalization.

The secretion of inflammatory mediators at the protein level was detected using a commercial enzyme-linked immunosorbent assay (ELISA) kit (R&D Systems, USA). Following culture on experimental surfaces for 3 and 7 days, specific media were harvested for cytokine quantification. The analysis measured M1-associated pro-inflammatory markers (IL-1β and TNF-α) and M2-associated pro-healing anti-inflammatory factors (PDGF-BB and IL-10) to evaluate macrophage functional phenotypes.

Osteoblasts were cultured on distinct substrate surfaces to detect the effects of GO modification and macrophage-conditioned medium on osteoblasts (Supplementary Figure 2B). For osteoblasts cultured on SLA/GO substrates with macrophage-conditioned medium, the designation SLA/GO + CM was applied. The cytoskeleton of osteoblasts was assessed through immunofluorescence staining techniques. After 4 days, the cells underwent fixation and membrane permeabilization using 0.1% Triton X-100, followed by successive incubation with FITC-conjugated phalloidin and DAPI nuclear stain (Sigma, USA). Fluorescence imaging was obtained employing a confocal laser scanning microscope (CLSM; Leica, Germany) for visualization and analysis.

On day 4 and 7, RT-PCR was performed to evaluate the expression of osteogenesis-associated markers in osteoblasts, including runt-related transcription factor 2 (RUNX2), alkaline phosphatase (ALP), osteopontin (OPN), and osteocalcin (OCN). Following a 24-h culture, osteoblasts grown on SLA/GO substrates were supplemented with macrophage-conditioned medium. The RT-PCR procedure adhered to the established protocol for macrophages.

To assess the mineralization ability, osteoblasts were cultured on the experimental substrates. The SLA/GO + CM group used macrophage CM, while the other groups were maintained in α-MEM. After 7 days, all the culture media were changed to osteogenic induction medium and the cultures were continued until day 21. Subsequently, the cells were fixed and stained with 0.1% Alizarin Red S (Sigma-Aldrich, USA) for 10 min to observe calcium deposition in the extracellular matrix. For quantitative analysis, 500 μL of 10% cetylpyridinium chloride was used to dissolve the mineralized nodules and the absorbance was measured at 600 nm.

### 2.7 *In Vivo* biological assessment

Twenty-seven 12-week-old male Sprague–Dawley (SD) rats were anesthetized by intraperitoneal injection of sodium pentobarbital (40 mg/kg body weight), resulting in a stable and deep anesthesia state that lasted approximately 1 h, sufficient for all experimental procedures to be completed. Before the surgery, a randomization sequence was generated using Microsoft Excel. Each rat was assigned to receive two implants (one SLA implant and one SLA/GO implant), with the left and right sides determined by the randomization sequence. All analyses after the surgery were conducted by researchers blinded to group allocation. The surgeons responsible for implant placement were also blinded to the surface modification of each implant until after the completion of all surgeries. The animal experiments were approved by the Animal Ethics Committee of the Tongji University School of Stomatology (approval no [2023]-DW-16) before they began.

The implant was first incubated in a bacterial solution (containing *P. gingivalis*, *F. nucleatum*, and *S. aureus*) at a concentration of 1 × 10^6^ CFU/mL for 24 h. Then, it was air-dried and subsequently surgically implanted. Following a 24-h post-implantation, the rats were euthanized, and the explanted implants were gently rolled across both LB and BHI agar plates to facilitate bacterial growth for 48 h (aerobic incubation to primarily recover *S. aureus*; anaerobic incubation to primarily recover *P. gingivalis* and *F. nucleatum*).

On day 7 post-implantation, the experimental rats were euthanized by intraperitoneal injection of an excessive dose of sodium pentobarbital (150 mg/kg body weight). The death was confirmed by the cessation of breathing and heartbeat, and absence of any reflexes. All operations were carried out in accordance with the AVMA Guidelines for the Euthanasia of Animals (2020 Edition). Then the femoral tissues were harvested. The collected femurs underwent fixation in 4% paraformaldehyde (PFA) for 2 days, followed by decalcification in a 10% EDTA solution (Sigma, USA) over a 60-day period, with the decalcifying agent refreshed every 48 h. Subsequently, the femoral specimens were subjected to graded ethanol dehydration and paraffin embedding. Using a microtome (Leica, Germany), 5 μm-thick histological sections were obtained.

For histological evaluation, half of the sections were stained with hematoxylin and eosin (H&E; Beyotime, China), and the remaining sections underwent immunofluorescence staining targeting Arg-1 to identify M2-polarized macrophages. Microscopic examination was performed using a Nikon FV1000 system. Inflammatory cell accumulation and Arg-1-positive cell ratios were quantified based on immune-histochemical staining results.

At postoperative intervals of 2, 4, and 8 weeks, femoral specimens containing implants were scanned applying micro-computed tomography (micro-CT) under standardized parameters. Bone volume fraction (BV/TV) and trabecular numerical density (Tb.N) were quantified employing the CTAn software suite (version 1.17; Skyscan, Kontich, Belgium). The analyzed region encompassed a 1.5 mm concentric zone surrounding the implant axis.

To evaluate peri-implant osteogenesis, Van Gieson (V-G) staining was performed on femoral specimens harvested at identical time points. Following fixation and dehydration, specimens were embedded in Technovit 7200 VLC resin (Heraeus Kulzer, Germany) and sectioned using a precision diamond saw (Exakt 300 C L, Germany). Subsequent refinement to approximately 50 μm thickness was achieved through sequential grinding and polishing (Exakt 400 CS, Germany). This is a hydrophilic resin; the sections can be directly stained without the need for prior deplasticization. Therefore, tissue sections were treated with V-G stain (Solarbio, China), wherein osteoid tissue exhibited characteristic red pigmentation. Bone-to-implant contact (BIC) percentages were subsequently calculated by ImageJ analysis.

### 2.8 Statistical analysis

All *in vitro* experiments were performed with at least three independent biological replicates (n = 3), each with three technical replicates; technical replicates were averaged and not treated as independent observations. The data were presented as the mean ± standard deviation (SD). Comparisons between two groups were performed using Student’s t-test; multiple groups were performed using one-way analysis of variance (ANOVA), followed by Tukey’s honestly significant difference (HSD) *post hoc* test. Before the statistical analysis, normality (Shapiro-Wilk test) and homogeneity of variance (Levene’s test) were confirmed. If the data deviated from normality, non-parametric tests were employed (Mann-Whitney U or Kruskal–Wallis test with Dunn’s *post hoc* test). If homogeneity of variance was violated, Welch’s ANOVA with Games-Howell *post hoc* test was used. For *in vivo* experiments, each animal received both SLA and SLA/GO implants (paired design); a paired t-test was performed at each time point, with each animal as the experimental unit. All analyses used SPSS 22.0 (IBM, USA). *P < 0.05* was considered statistically significant.

## 3 Results and discussion

### 3.1 Surface characterization of GO-Engineered titanium substrates

In this investigation, the GO with nanostructures (Supplementary Figure 3) was deposited on the titanium substrate by using ultrasonic spraying technology. This method has previously been proven to be an excellent approach for nanomaterial modification (Hergli et al., 2024). The GO suspension was atomized through high-frequency ultrasonic oscillation, and relative to conventional approaches like drop-casting and immersion techniques, this spraying method offers distinct benefits including: (1) efficient deposition rate, (2) uniform dispersion and controlled orientation, (3) high processing capacity, and (4) precise thickness regulation.

After surface modification, surface morphology of the substrate was captured by a SEM. As shown in Figure 1A, the titanium substrate presented an irregular and rough surface after SLA treatment, with randomly distributed pits of varying dimensions on it. Similarly, at a lower magnification, the SLA surface engineered with GO also retained this similar pit-like morphology. In high-resolution imaging, obvious wrinkled structures were observed on the sample surface, which is commonly referred to as asperities in the literature (Han et al., 2022). This characteristic morphology confirmed the successful deposition of GO, thereby forming a wrinkled surface structure (Qiu et al., 2017).

**FIGURE 1:**
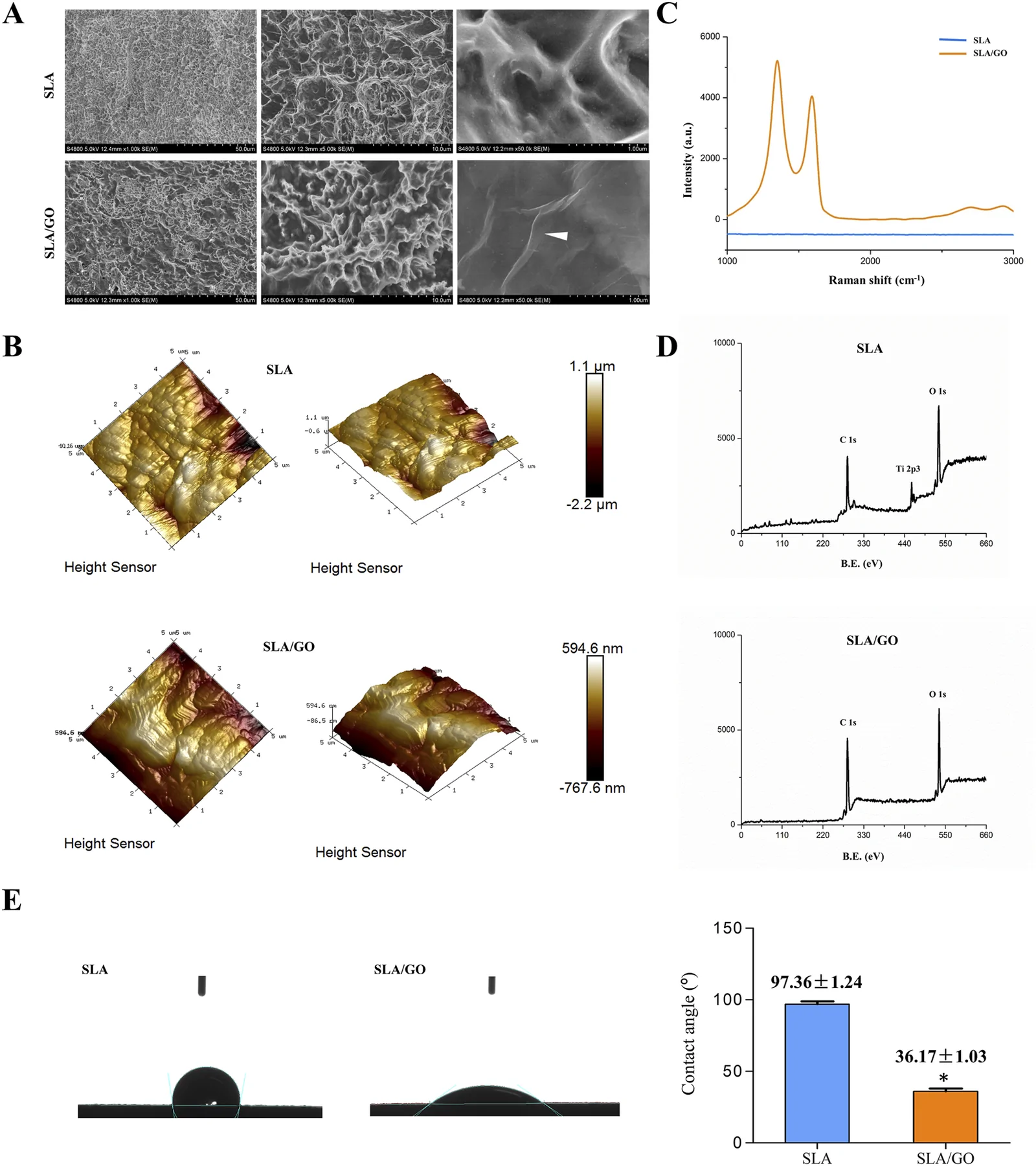
Comparative analysis of SLA and SLA/GO-modified substrates **(A)** Surface morphologies exhibited by SEM; wrinkle-like GO features are indicated by arrow **(B)** AFM-assessed nanoscale topography **(C)** Chemical structure analysis via Raman spectroscopy **(D)** Elemental composition from XPS survey scans **(E)** Wettability assessment. *p < 0.05 versus the SLA control.

The topography was further characterized using AFM, with detailed microstructural features presented in Figure 1B. Quantitative analysis revealed significant differences in roughness parameters between SLA and SLA/GO samples. Specifically, the SLA surface exhibited higher roughness values, with an average roughness (Ra) of 235.87 ± 4.62 nm and a root-mean-square roughness (Rq) of 309.21 ± 9.87 nm. In contrast, the GO-engineered surface demonstrated substantially lower Ra (98.34 ± 5.69 nm) and Rq (126.37 ± 1.46 nm) values. According to the literature, an implant with a surface roughness ranging from 100 to 150 nm was identified as the optimal surfaces for achieving excellent bone formation (Wennerberg and Albrektsson, 2010). Furthermore, surface roughness affects macrophage polarization and induces macrophages into a specific material-activated state, which only a very narrow range of rough surfaces (with Ra values ranging from 51 to 136 nm) would induce macrophages to polarize into the M2 phenotype (Zhang et al., 2019). This GO-engineered surface, with reduced roughness, may be partially beneficial for bone formation and macrophage polarization to the M2-skewed phenotype in our investigation.

Characterization of the GO modification was confirmed by Raman spectroscopy (Figure 1C). Distinctive peaks, namely, the D band at ∼1,350 cm^-1^ and the G band at ∼1,580 cm^-1^, were observed on the GO-engineered surface, corresponding to the structural features of GO. The D band reflects the defect level and crystallinity, whereas the G band arises from the sp^2^ graphitized structure, in agreement with prior studies (Jeong et al., 2016). Notably, these characteristic peaks were absent on the SLA surface, also confirming the successful deposition of GO while preserving its intrinsic structure after spraying. Additionally, XPS analysis revealed the presence of C, O, and Ti on the SLA modified surface (Figure 1D). In contrast, no Ti signal was detected after GO modification, indirectly suggesting complete and uniform coverage of the GO on the substrate.

The wettability of the surface was assessed employing contact angle measurements (Figure 1E). Following GO modification, a notable reduction in contact angles was observed (SLA: 97.36° ± 1.24°; SLA/GO: 36.17° ± 1.03°), demonstrating enhanced hydrophilicity after GO modification. The surface hydrophilicity facilitates the adsorption of extracellular matrix (ECM) proteins, thereby promoting cell proliferation, adhesion, differentiation, and subsequent bone mineralization (Velasco-Ortega et al., 2023). This improvement in wetting property is attributed to the abundant hydrophilic oxygen functional groups, such as carboxyl (−COOH) and hydroxyl (−OH), present in the GO structure. Moreover, studies have shown that compared to hydrophobic surfaces, hydrophilic titanium surfaces would trigger a milder inflammatory response (Donohoe et al., 2023). Specifically, macrophages cultured on highly hydrophilic substrates tend to form an anti-inflammatory microenvironment, thereby enhancing the osseointegration of biomaterials (Hotchkiss et al., 2016). In addition to regulating the immune response, such surfaces can also enhance osteogenic activity, promoting the differentiation of osteoblasts and bone marrow mesenchymal stem cells (BMSCs) both *in vitro* and *in vivo* (Jacobs et al., 2024). Therefore, enhancing the hydrophilicity of the material not only reduces inflammation but also has a positive impact on osteogenesis, indicating that GO modification plays a very effective role in the surface engineering of implants.

### 3.2 *In Vitro* antibacterial performance

The bacteria *P. gingivalis* and *F. nucleatum* have been confirmed as key pathogens causing peri-implant infections (Heyman et al., 2022; Chen et al., 2022). Additionally, *S. aureus* exhibits significant adhesion ability on titanium surfaces and plays a major role in the development of peri-implantitis (Zhang et al., 2023). Due to the clinical relevance of these three bacteria, this study selected them to evaluate antibacterial properties. Figure 2A shows a photo of bacterial colonies on the dish, and Figure 2B presents the corresponding quantitative count results. Compared with the SLA, the GO engineered surface shows fewer bacterial colonies and enhanced antibacterial activity. Supplementary Figure 4 analyzes the antibacterial effects of the SLA surfaces and the GO modified surfaces on *P. gingivalis*, *F. nucleatum*, and *S. aureus*. Moreover, the bacteria were cultured on the substrate for 12 h and then stained with fluorescence. As shown in Figure 2C, live bacteria are marked with green fluorescence, while dead bacteria are marked with red signals. It is notable that the GO engineered surface shows extensive red fluorescence, while the control group mainly shows green signals. These findings further confirm that the SLA/GO surface has antibacterial activity, which is consistent with the above experimental result.

**FIGURE 2:**
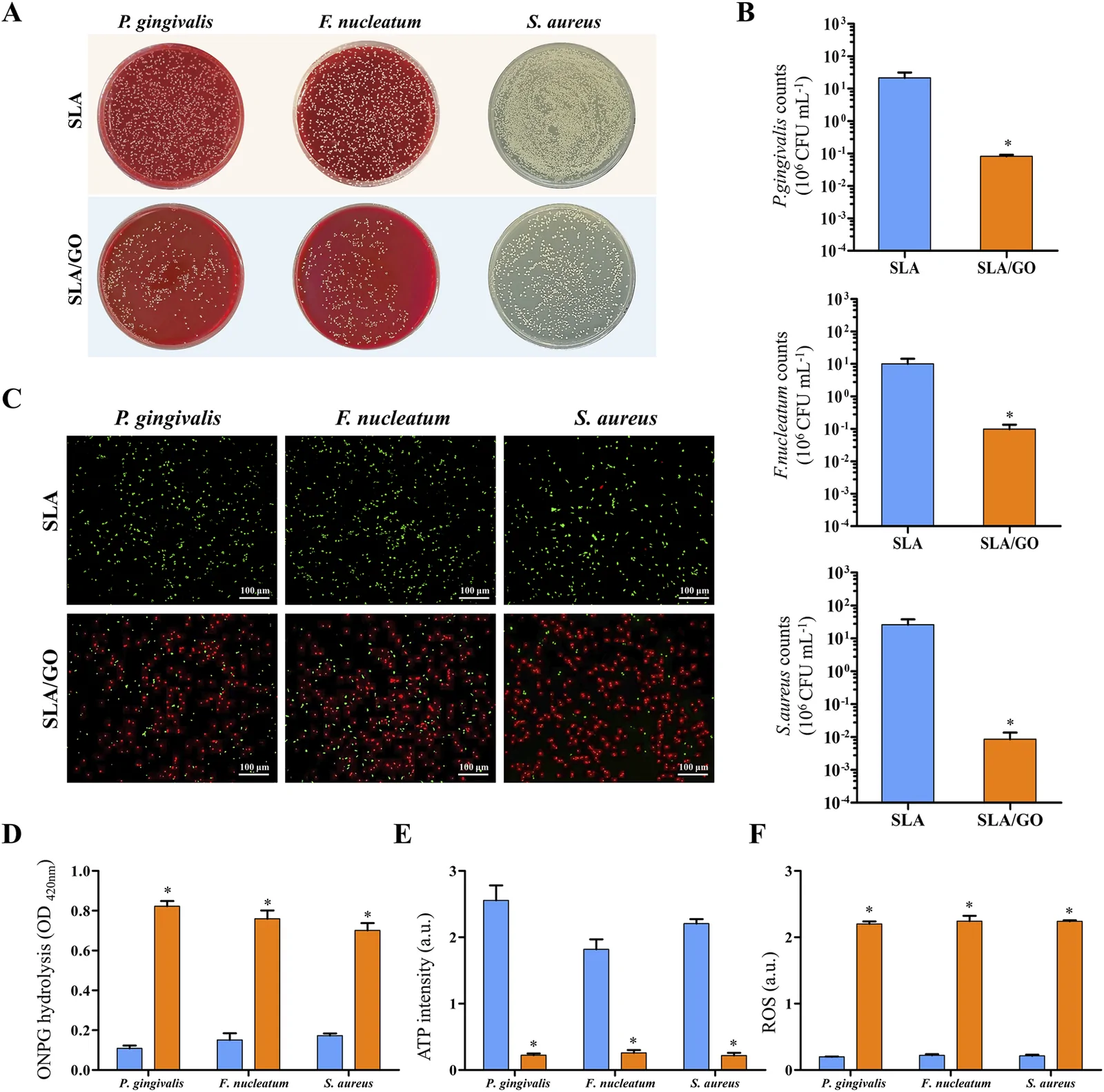
Antibacterial activity of the SLA and SLA/GO modified substrates **(A)** Photographs of bacterial colonies grown on plates after 2 h of direct contact with SLA and SLA/GO modified substrates **(B)** Quantitative data of colony counts of samples toward *Porphyromonas gingivalis*, *Fusobacterium nucleatum*, and *S. aureus* **(C)** Viable bacteria staining images: green fluorescence was observed in viable bacteria, whereas red fluorescence was assigned to non-viable cells **(D)** ONPG hydrolysis degree and **(E)** ATP intensity of bacteria on SLA and SLA/GO modified substrates **(F)** Intracellular ROS production of bacteria cultured on SLA and SLA/GO modified substrates. *p < 0.05 versus the SLA control.

As cell membrane permeability rises, ONPG diffuses into the intracellular space, where it is hydrolyzed by β-galactosidase to generate ortho-nitrophenol. The concentration of this product can be quantified by assessing the optical density at 420 nm (OD_420_) (Zhang et al., 2022). Based on this principle, membrane permeability was evaluated using the ONPG assay. Figure 2D demonstrates that SLA/GO promoted higher ONPG hydrolysis activity compared to the SLA surface, suggesting enhanced membrane permeability in bacteria cultured with SLA/GO. Additionally, bacterial cultures exposed to SLA/GO showed reduced adenosine triphosphate (ATP) levels (Figure 2E), further confirming the decline in ATP metabolic activity. The generation of ROS in bacteria was detected using the 2′,7′-dichlorofluorescein diacetate probe. Figure 2F shows that the intracellular ROS contents of bacteria cultured on SLA/GO increased compared with those of bacteria on the SLA surface. According to a previous study, the oxidative stress caused by GO’s oxygen-containing functional groups helps to release ROS, which eventually results in ROS-mediated bacterial apoptosis (Yao et al., 2020). In concordance with these findings, our results showed that GO to resist bacterial infections may be related to its ability to increase bacterial membrane permeability and promote the generation of ROS within the bacteria.

Previous studies have provided explanations for the antibacterial mechanism of GO. The antibacterial property mainly lies in its ability to directly damage the cell membrane, thereby achieving an inhibitory effect (Huang et al., 2020; Pulingam et al., 2021). Investigations examining GO nanosheets’ impact on *E. coli* membranes have identified dual mechanistic pathways. The first one involves physical penetration of bacterial membranes by the nanomaterial’s sharp edges, while the second entails lipid extraction through hydrophobic interactions between GO sheets and membrane phospholipids, ultimately causing structural damage (Akhavan et al., 2011; Tu et al., 2013). Furthermore, studies on GO-cobalt ferrite nanocomposites (GO-CFNCs) demonstrated that GO components disrupt protein translocation and membrane assembly processes. This interference occurs through specific interactions with threonine (THR) and glycine (GLY) amino acid residues via hydrogen bonding and hydrophobic forces. Although the reported mortality rate was only 19.6% for *E. coli* and 81.6% for *S. typhimurium*, the nanocomposites were also found to penetrate bacteria and impair the activity of oxidoreductase enzymes that mitigate oxidative stress, as evidenced by the detection of intracellular ROS (Arun et al., 2019).

Comparative studies have highlighted the differential antibacterial mode of GO against Gram-positive and Gram-negative strains. Microscopic analysis showed that Gram-positive bacteria cultured with GO predominantly exhibited cellular entrapment, whereas Gram-negative strains suffered more pronounced membrane disintegration through direct physical interaction with the GO (Deokar et al., 2013). These findings underscore the structure-dependent antibacterial behavior of GO across bacterial classifications. In our experiment, it was confirmed that the GO modification has an antibacterial effect on the bacteria related to peri-implant infections. The speculated mechanism is to enhance the permeability of the bacteria and the level of ROS within the bacteria. Thus, how GO disrupts the permeability of bacteria, whether its effects on the above three types of bacteria are different, and what molecular changes occur following the disruption of bacterial permeability, will be the key topics for our future research.

In addition, compared with some advanced antibacterial implant coatings, the implant coated with GO in this study (with an antibacterial efficiency of approximately 70%) is at a moderate level. For instance, quaternized chitosan-based coatings on Ti or Ti-6Al-4V (TC4) surfaces have demonstrated strong antibacterial activity exceeding 90% against common pathogens, including *E. coli*, methicillin-resistant *S. aureus* (MRSA), and oral anaerobic bacteria such as *P. gingivalis* and *F. nucleatum*. Phloretic acid-modified quaternized chitosan (PQCS)-Ti also exhibits potent antibacterial activity against *P. gingivalis* and *F. nucleatum* (Chen et al., 2025; Xie et al., 2027). The excellent performance of these coatings is mainly attributed to the membrane disruption mechanism of quaternized chitosan: the positively charged quaternary ammonium groups interact with the negatively charged bacterial membrane, causing physical membrane disruption and bacterial death. In contrast, our GO coating exerts its antibacterial effect through ROS-induced oxidative stress and mechanical membrane damage. However, compared with the potent membrane disruption effect of quaternized chitosan, the degree of oxidative stress is relatively mild, and the antibacterial activity is highly dependent on the direct contact between the GO and the bacteria, thus resulting in an intermediate effect. These findings highlight the limitations of the GO coating and indicate that future strategies involving antibacterial polymers (such as quaternized chitosan) with GO can be adopted to achieve enhanced and synergistic antibacterial effects.

### 3.3 *In Vitro* osteoimmunomodulatory performance

As the primary immune cells encountering the implant surface upon insertion, macrophages significantly influence the process of bone-implant integration (Li et al., 2022a). An overabundance of M1-polarized macrophages around orthopedic implants is associated with bone resorption and implant failure, whereas promoting the anti-inflammatory M2 phenotype is beneficial for osseointegration (Rao et al., 2012). Consequently, modulating polarization of macrophage toward the anti-inflammatory and pro-regenerative M2 phenotype is vital for enhancing bone repair and implant osseointegration. Alterations in implant surface characteristics, such as topography and roughness, can indirectly affect macrophage behavior, including adhesion, viability, gene regulation, and cytokine secretion (Pitchai et al., 2022; Li et al., 2022b). These findings highlight the sensitivity of macrophages to specific surface modifications, underscoring the importance of optimizing nanoscale surface engineering to facilitate osseointegration via macrophage polarization (M1/M2).

To investigate the biosafety of the GO coating, the apoptosis of macrophages grown on the substrates was assessed by FCM as shown in Supplementary Figure 5. Annexin V^−^PI^-^ (the fourth quadrant) represents live cells, Annexin V^+^PI^−^ (the third quadrant) represents early apoptotic cells, and Annexin V^+^PI^+^ (the second quadrant) represents late apoptotic cells. There was no significant difference in cell survival rate or apoptosis rate between SLA and SLA/GO at 1, 3, and 7 days after inoculation. Therefore, GO modification has no toxic effect on macrophage survival and apoptosis.

As demonstrated in Figure 3A, after 3 days of culture, GO-modified titanium substrates exhibited higher percentages of CD163^+^ and CD206^+^ (M2-associated) macrophages compared to SLA controls. Conversely, SLA surfaces showed greater levels of CD86^+^ and CD11c^+^ (M1-associated) macrophages (Figure 3C). After 7 days (Figures 3B,D), the same polarization pattern was maintained, and notably, M2-skewed phenotype polarization on GO-modified surfaces progressively increased over time, whereas SLA surfaces did not show this trend. These flow cytometry data indicate that GO functionalization preferentially promotes an anti-inflammatory M2-skewed macrophage phenotype.

**FIGURE 3:**
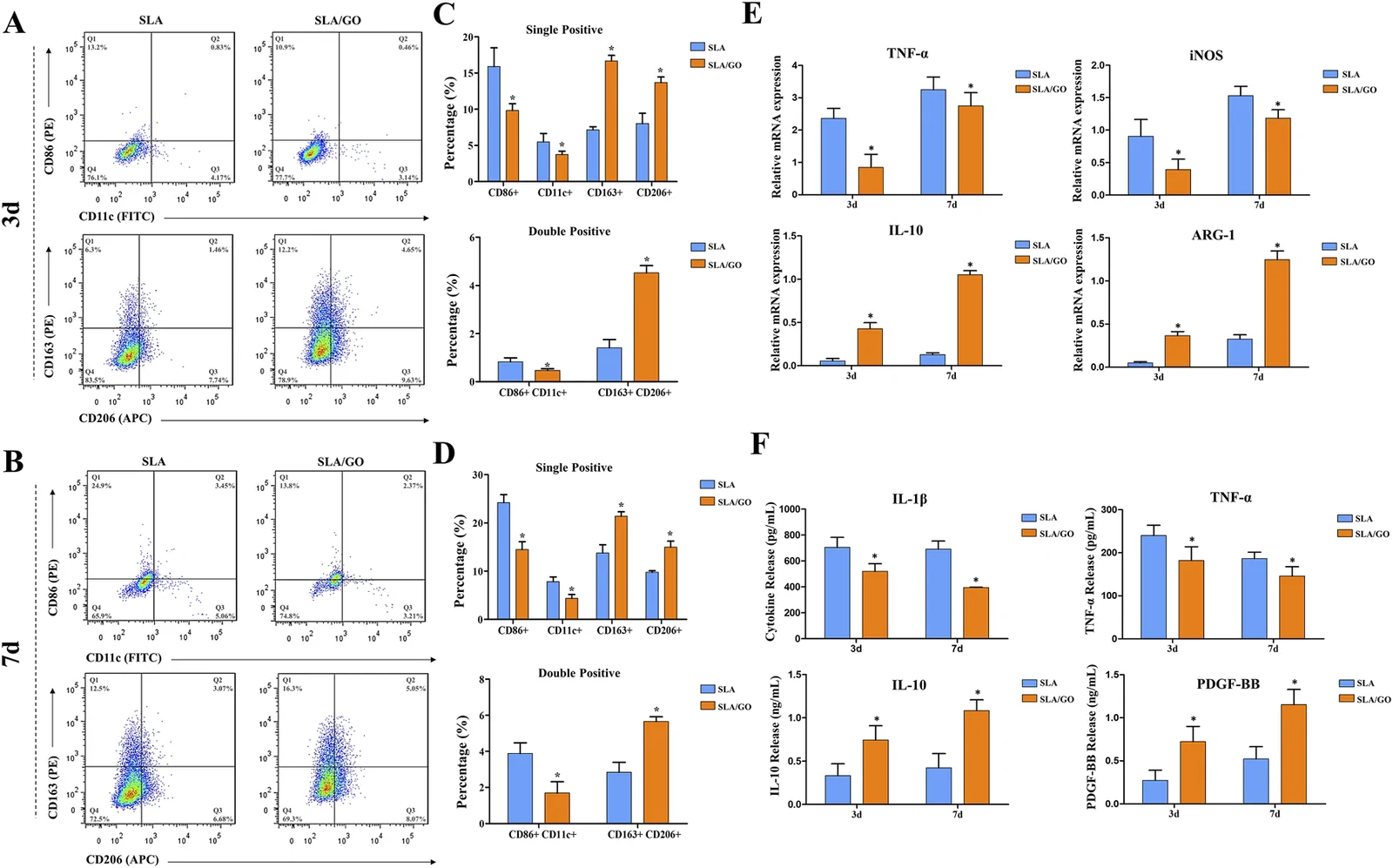
Macrophage phenotype analysis on SLA and SLA/GO modified substrates **(A, B)** Flow cytometric analysis of specific surface markers at 3 and 7 days **(C, D)** Proportions of single-positive (CD11c^+^, CD86^+^, CD206^+^, CD163^+^) and double-positive (CD86^+^CD11c^+^, CD163^+^CD206^+^) populations at the respective time points **(E)** Relative mRNA expression levels determined by RT-PCR **(F)** Cytokine secretion profiles measured by ELISA. *p < 0.05 versus the SLA control.

Quantitative RT-PCR analysis (Figure 3E) supported these findings. Macrophages cultured on SLA surfaces expressed higher levels of M1-related genes (iNOS and TNF-α) at both 3 and 7 days compared to those on SLA/GO substrates. In contrast, the SLA/GO surface showed markedly elevated expression of M2-related genes (Arg-1 and IL-10). Consistent with this, cytokine measurements (Figure 3F) revealed that GO modification reduced the secretion of pro-inflammatory cytokines (TNF-α, IL-1β) while increasing anti-inflammatory (IL-10) and pro-healing (PDGF-BB) factors. Collectively, these data demonstrate that surface-immobilized GO promotes M2-skewed phenotype polarization in macrophages. Our flow cytometric analysis did not include a pan-macrophage marker such as F4/80 to identify the total macrophage population. Therefore, we cannot completely rule out the possibility that the selective inhibition of M1 macrophages or preferential survival or promotion of M2 macrophages, may partially contribute to the observed effects. However, the progressive increase in M2-skewed polarization on the GO modified surface from day 3 to day 7 supports the view that GO promotes M2-skewed phenotype polarization. This explanation is also supported by RT-PCR and cytokine secretion results. Future studies incorporating F4/80 staining will help to clearly distinguish the relative contributions of active M2 polarization, M1 inhibition, or M2 subsets selective survival.

We further explored potential surface properties that could account for this immunomodulatory effect. Physical characterization revealed that GO deposition altered the original SLA morphology, generating nanoscale wrinkling and reducing surface roughness. Previous studies have shown that nanostructured surfaces tend to suppress inflammatory cytokine release compared to micro-structured surfaces (Luu et al., 2015; Lee et al., 2011). In line with these observations, our GO-modified surface—with its nanoscale features—induced lower pro-inflammatory mediators than the micro-rough SLA surface. Additionally, the extreme hydrophilicity of the GO-engineered surface has been reported to promote M2 polarization through integrin-mediated signaling (Korakaki et al., 2023; Lv et al., 2018). Thus, the combination of nanoscale topography and high wettability may result in the observed immunomodulatory effects of our SLA/GO substrate. Importantly, the M2-associated cytokines (e.g., IL-10) are known to enhance MSCs proliferation and osteogenic differentiation, whereas M1-associated cytokines (e.g., IL-1β, TNF-α) suppress them (Xu et al., 2020). Therefore, the GO-induced M2-skewed phenotype polarization observed in this study is expected to create an immunomodulatory microenvironment that facilitates bone-implant integration. The precise molecular mechanisms underlying GO-mediated macrophage polarization—such as the possible involvement of specific signaling pathways (NF-κB, MAPK, STAT3/6) or phagocytic recognition—were not investigated here and these pathways represent plausible hypotheses warrant further study (Liu et al., 2020; Overlin et al., 2020; Li et al., 2021; Linton et al., 2019; Li et al., 2019).

It is worth noting that our current experimental design did not include a control group to distinguish the individual contributions of the nanostructures and enhanced hydrophilicity to the observed M2-skewed polarization. Therefore, although the immunomodulatory effect may be attributed to the comprehensive signals provided by GO modification, namely, the reduction in surface roughness and the improvement in wetting properties, we cannot distinguish these two factors. This constitutes a limitation of this study. Future research that can introduce systematic decoupling of these factors, such as using a smooth GO coating or a hydrophobic GO surface, will help to further clarify the relative contributions of each surface property.

Traditional *in vitro* evaluation of osteoinductive biomaterials relies heavily on osteogenic lineage cells, yet the suboptimal clinical performance of some materials suggests that successful osseointegration requires multi system coordination. Emerging evidence highlights the critical role of macrophages and their crosstalk with other cells. We therefore propose that surface modification may modulate macrophage polarization, thereby influencing osteoblast function.

To test this, we used macrophage conditioned medium (CM) to culture osteoblasts. As shown in Figure 4A, osteoblasts exposed to medium from macrophages on SLA/GO surfaces exhibited enhanced spreading and cytoplasmic extension compared to those on SLA or SLA/GO surfaces directly. Quantitative RT-PCR (Figure 4B) revealed that the CM from the SLA/GO surface upregulated osteogenic gene expression (RUNX2, ALP, OCN, and OPN) after 4 and 7 days. Moreover, after 21 days of culture in the CM, matrix mineralization (Figure 4C) was greatest in SLA/GO conditioned medium group (*p < 0.05*). These findings demonstrate that the CM from the macrophages cultured on the GO modified group more effectively stimulates osteogenic differentiation of osteoblasts, consistent with the observed M2-skewed polarization. We did not conduct direct Western blot verification of the expression of osteogenic-related proteins. But, our RT-PCR results showed significant upregulation of related genes at the transcriptional level, and the Alizarin Red S staining confirmed that the matrix mineralization was enhanced at the functional level; future Western blot analysis of osteogenic proteins would help further confirm our results.

**FIGURE 4:**
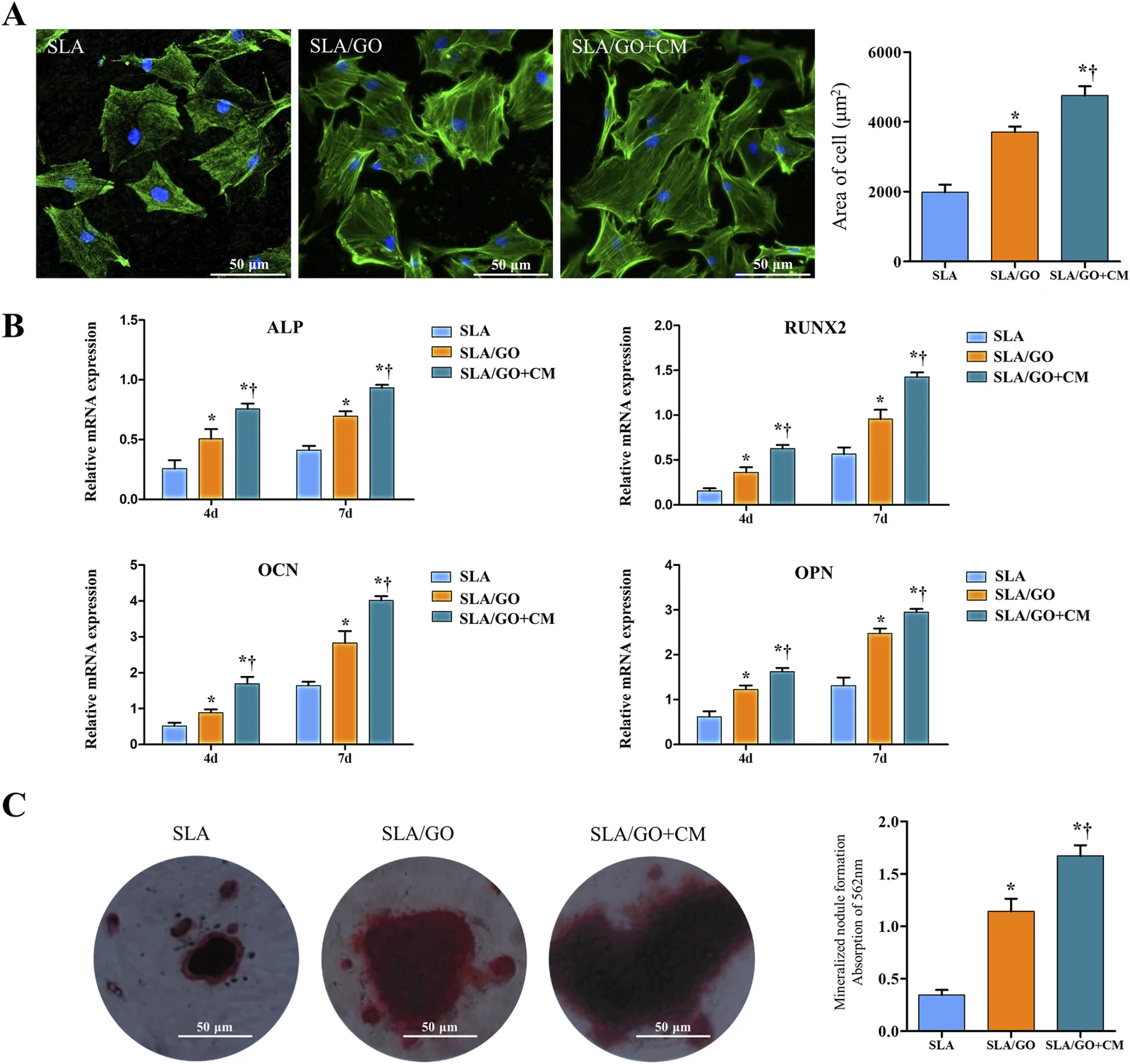
Osteogenic results of osteoblasts to macrophage-CM **(A)** Morphology of osteoblasts on SLA, SLA/GO surfaces and SLA/GO + CM **(B)** mRNA expression levels of osteogenic genes in osteoblasts cultured on SLA, SLA/GO surfaces and SLA/GO + CM for 4 and 7 days, analyzed by RT-PCR **(C)** Mineralized matrix deposition after 21 days of culture in the CM, with corresponding quantitative analysis. *p < 0.05 versus the SLA; ^*†*^ *p < 0.05 versus the SLA/GO*.

Previous studies have indicated that culture supernatant from M2-type macrophages can enhance the transcription of osteogenic differentiation-related genes and facilitate mineral deposition in the extracellular matrix (Chen et al., 2016). Furthermore, cytokines secreted by anti-inflammatory M2 macrophages promote MSCs functions, whereas those produced by pro-inflammatory M1 macrophages suppress MSC activities *in vitro* (Hu et al., 2023; Murayama et al., 2024). These observations align well with our results. In the present study, the SLA/GO group exhibited enhanced M2-skewed macrophage polarization compared to the SLA group, associated with elevated anti-inflammatory cytokine production and reduced pro-inflammatory mediator levels. Consequently, the CM derived from macrophages on the SLA/GO surface showed the strongest ability to enhance osteogenic gene expression in osteoblasts and matrix mineralization.

Additionally, GO modification improved osteoblast spreading and promoted their osteogenic gene expression and matrix mineralization, outperforming the conventional SLA surface. While GO modification altered surface topography, roughness, and wettability—factors known to influence cell behavior—we did not directly isolate these parameters in this study. The precise mechanisms underlying the enhanced osteogenic response remain to be elucidated. Nevertheless, our results clearly demonstrate that GO-functionalized surfaces create a microenvironment more favorable for osteoblast function, leading to stronger pro-osteogenic effects compared to SLA surfaces.

### 3.4 *In Vivo* antibacterial efficacy and immune-osteogenic performance

Achieving stability and favorable outcomes for dental implants is significantly hindered by the risk of peri-implant bacterial colonization. Despite the implementation of rigorous sterilization protocols in surgical procedures, the inherent non-sterile nature of the oral environment creates substantial potential for microbial contamination (Wang et al., 2022). Existing titanium-based implant materials exhibit limited antimicrobial activity, and research indicates that diverse bacterial communities rapidly colonize on the implant surface after surgical placement. Following initial colonization, these bacteria promote the attachment of additional microbial populations, accelerating biofilm development and substantially elevating the probability of peri-implantitis complications (Fernandes et al., 2024). Therefore, excellent implants possess their own antibacterial properties. In this study, on the first postoperative day, the implanted materials were retrieved and subsequently rolled onto culture plates for incubation over a 48-h period. As illustrated in Figure 5A, the SLA-treated samples exhibited extensive bacterial colonization, with dense clusters merging into confluent growth. In contrast, the SLA/GO-modified implants demonstrated a marked reduction in viable bacterial counts. We note that this study employed a pre-incubation contamination model (where the implant was co-cultured with bacteria for 24 h before implantation), rather than a true *in vivo* infection model. This is a limitation of our study. Therefore, our subsequent investigations will adopt a ligature-induced peri-implantitis model, which more closely reflects the clinical progression of peri-implant inflammation.

**FIGURE 5:**
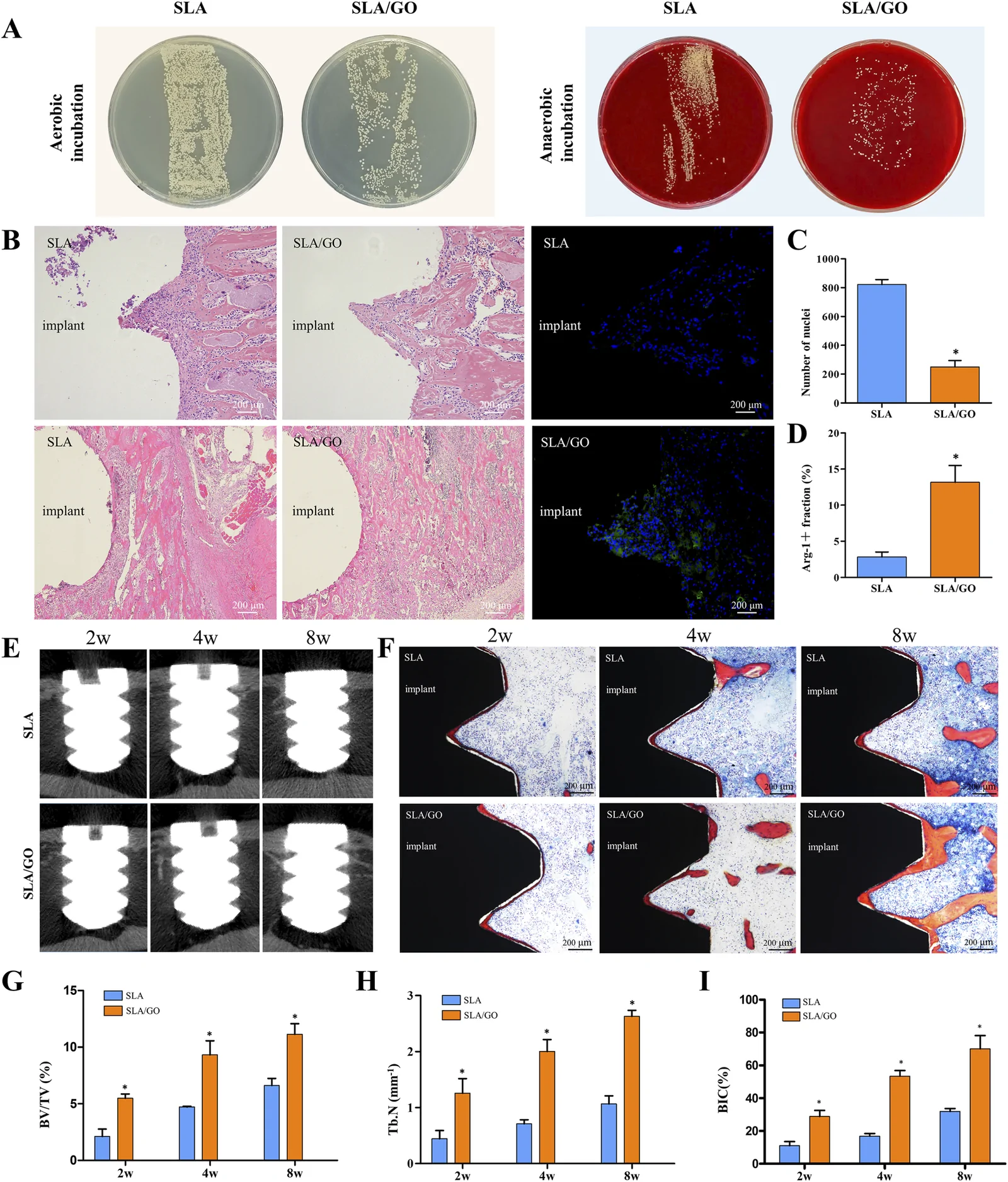
*In vivo* performance of SLA and SLA/GO surface-modified implants **(A)** Antibacterial effect assessed by culturing aerobic and anaerobic bacteria **(B)** Local inflammatory response characterized by H&E staining and immunofluorescence for Arg-1^+^ macrophages **(C, D)** Subsequent quantification of inflammatory cell density and Arg-1^+^ macrophage percentage **(E, F)** Osseointegration monitored over time via micro-CT imaging and V-G staining at 2, 4, and 8 weeks **(G, H)** Quantitative analysis of BV/TV and Tb.N. **(I)** Histomorphometric evaluation of bone-implant contact (BIC). *p < 0.05 versus the SLA control.

Following implantation, macrophage accumulation occurs at the material-tissue interface, triggering localized inflammatory reactions (Zhao et al., 2022). Therefore, in the present study, inflammatory cell distribution surrounding the implant served as an indicator of inflammatory intensity, while Arg-1-expressing macrophages was used to evaluate M2-skewed polarization status (Figure 5B). Histological analysis revealed distinct differences between groups: SLA titanium implants exhibited extensive inflammatory cell aggregation at 7 days post-implantation, whereas SLA/GO implants demonstrated markedly reduced inflammatory infiltration. Besides, comparative analysis (Figure 5C) revealed attenuated inflammatory reactions in the SLA/GO implants compared to SLA controls, demonstrating GO’s anti-inflammatory properties in this experimental model. Furthermore, as demonstrated in Figure 5D, the GO-modified implants exhibited a markedly higher ratio of M2-skewed phenotype macrophages compared to SLA implants. Collectively, these findings indicate that the incorporation of GO enhances the modulation of inflammatory responses at the bone-implant interface, facilitating a shift from inflammation to tissue repair through M2-skewed macrophage polarization.

Through radiographic scanning, 3D reconstruction and subsequent quantitative analysis, the bone volume within the implant thread area was quantified. The presented data included the original image results (Figure 5E) and calculated parameters, specifically the BV/TV and Tb.N (Figures 5G,H). Comparative analysis showed that at the observation points of 2, 4 and 8 weeks, the implants modified with the SLA/GO surface had higher bone volume and trabecular density than the traditional SLA modified surface (*p < 0.05*). To evaluate osteogenic performance, histological and quantitative analysis of the BIC was conducted (Figures 5F,I). At 2 weeks post-operation, new bone was visible in all experimental groups (indicated by red staining), but the BIC value of the SLA/GO group was higher than that of the SLA surface (*p < 0.05*). This trend persisted at 4 and 8 weeks, and the SLA/GO implants consistently outperformed the SLA control group in BIC and new bone deposition. This finding indicates that GO promotes bone formation around the implant during the early healing stage. The enhanced bone formation observed is likely the result of multiple factors working together: in addition to stimulating osteoblast differentiation, GO also exerts immunomodulatory effects by regulating macrophage phenotypes, collectively contributing to its osteogenic properties. In summary, the results of the animal experiments highlight the important role of GO-modified implant surfaces in accelerating inflammation resolution, shortening osseointegration time, and achieving initial stability. We note that the *in vivo* observation period of this study was limited to 8 weeks after implantation. Although this time point has been widely used in rodent models to assess early osseointegration and initial healing of the bone-implant interface (Xu et al., 2022), it is not sufficient to evaluate the long-term effects of GO-modified implants. The 8-week observation period enables us to capture the *in vivo* changes in critical early stages such as bone formation and M2-skewed macrophage polarization, which are the main focus of this study. However, to evaluate chronic inflammation, delayed implant loosening, and the long-term biocompatibility of GO, a longer observation period is required. Future studies that include long-term follow-up are necessary to further confirm the clinical safety and durability of GO-modified dental implants. Moreover, this study mainly focuses on local tissue responses and osteogenic effects. Although the results of *in vivo* implantation indicated good local biocompatibility, no systematic safety evaluation including histopathological examinations of major organs (heart, liver, spleen, lungs, kidneys) and assessment of systemic toxicity markers was conducted. This is a limitation of this study. Future research will incorporate these evaluations to more comprehensively elucidate the safety characteristics of the GO coating, which is crucial for its potential clinical application.

## 4 Conclusion

This research presents a novel titanium implant functionalized with GO, designed to prevent bacterial infection and enhance osseointegration through immunomodulation (Figure 6). The GO-modified surface demonstrated antibacterial properties by disrupting microbial membranes and increasing ROS generation within bacteria. *In vitro* studies revealed that the GO-modified surface not only stimulated polarization of the M2-skewed macrophage phenotype, which promotes tissue repair, but also improved the extension and differentiation of osteoblasts by modulating the surrounding immune environment. Animal experiments provided further evidence that the GO reduced bacterial colonization in the pre-contamination model, accelerated inflammation resolution, and enhanced osseointegration. These findings indicate that GO modification represents a promising preclinical strategy to address compromised osseointegration resulting from bacterial colonization and inflammation associated with macrophage polarization. By simultaneously incorporating antibacterial and osteoimmunomodulatory functions, this study introduces an innovative approach to implant surface engineering. The multi-functional modification holds substantial promise for preclinical applications in both dental and orthopedic fields, potentially improving the success of medical implants.

**FIGURE 6:**
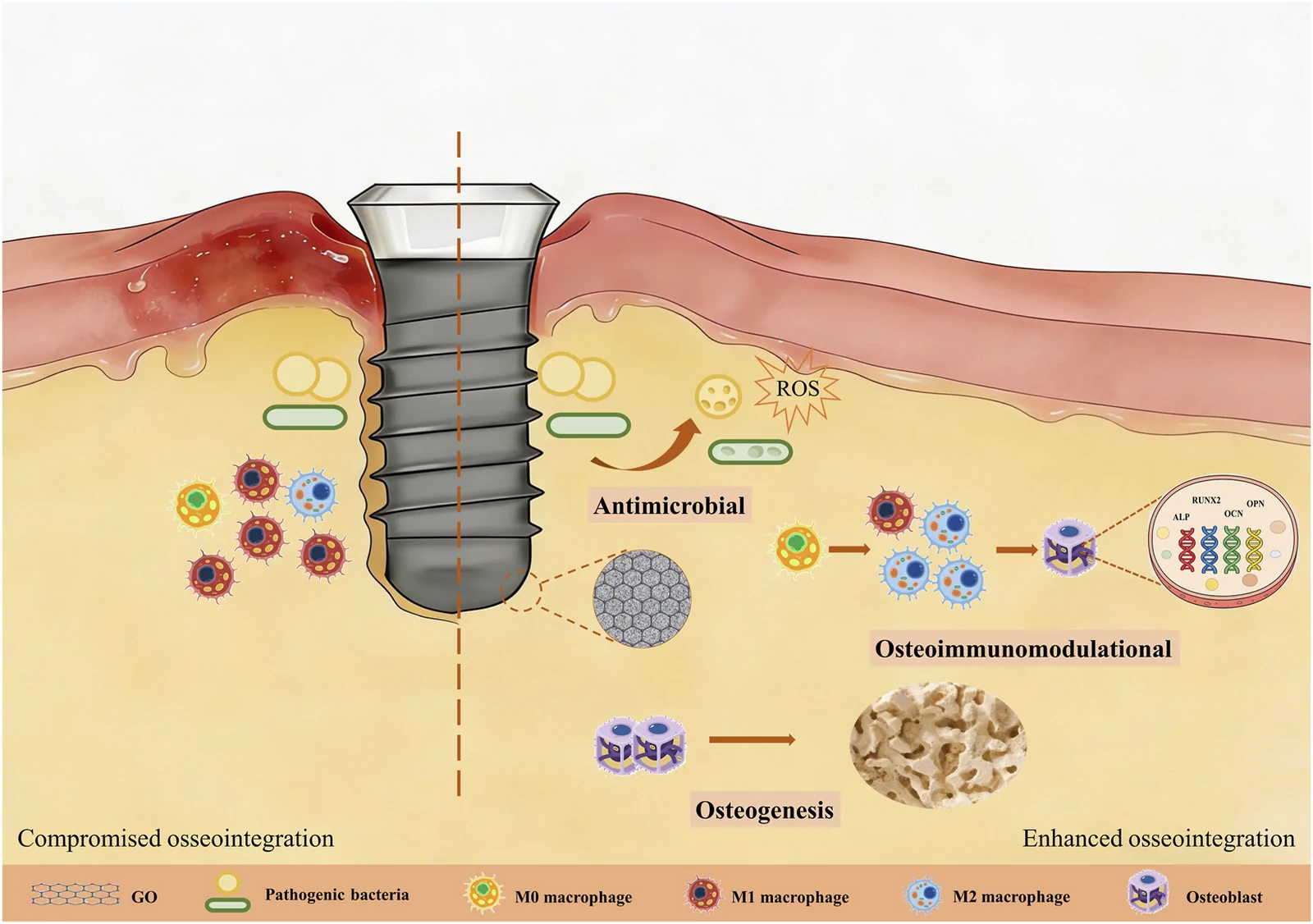
A novel titanium implant functionalized with GO to prevent bacterial infection and enhance osseointegration through immunomodulation.

## Data Availability

The original contributions presented in the study are included in the article/Supplementary Material, further inquiries can be directed to the corresponding author.
