# Supplementary material for: Graphene oxide-engineered dental implants: orchestrating antibacterial defense and osteo-immunomodulation for enhanced osseointegration
Source: Front Bioeng Biotechnol. 2026 Sep 23;14:1943704. doi: 10.3389/fbioe.2026.1943704 (PMC13642026; doi:10.3389/fbioe.2026.1943704)
Supplement: Supplementary file 1 [file DataSheet1.docx]

Supplementary Material

# 1 Supplementary Figures

# Supplementary Figure 1. The digital photograph of titanium substrate modified by SLA, SLA/GO and dental implant modified by SLA, SLA/GO.

# Supplementary Figure 2. Transmission electron microscopy scanning of the nano-structure of GO.

# Supplementary Figure 3. Schematic illustration of the culture models used to assess the direct effects of GO-modified substrates on macrophages and osteoblasts, and the indirect effects of macrophage-conditioned medium on osteoblasts.

# Supplementary Figure 4. The antibacterial efficacy rates of SLA and SLA/GO surface against *P. gingivalis*, *F. nucleatum*, and *S. aureus* respectively.

# Supplementary Figure 5. Survival status of macrophages on the SLA and SLA/GO surfaces was analyzed by flow cytometry at 1, 3, and 7 days.
